# Low-dose ticagrelor yields an antiplatelet efficacy similar to that of standard-dose ticagrelor in healthy subjects: an open-label randomized controlled trial

**DOI:** 10.1038/srep31838

**Published:** 2016-08-24

**Authors:** Pan Li, Ying Gu, Yawei Yang, Lizhi Chen, Junmei Liu, Lihong Gao, Yongwen Qin, Quancai Cai, Xianxian Zhao, Zhuo Wang, Liping Ma

**Affiliations:** 1 Department of Cardiology, Changhai Hospital, Second Military Medical University, 168 Changhai Rd, Shanghai, 200433, China; 2 Department of Pharmacy, Changhai Hospital, Second Military Medical University, 168 Changhai Rd, Shanghai, 200433, China; 3 Center for Clinical Epidemiology and Evidence-based Medicine, Second Military Medical University, 168 Changhai Rd, Shanghai, 200433, China

## Abstract

Ticagrelor has a greater antiplatelet efficacy than clopidogrel but may be accompanied by an increased risk of bleeding. This study evaluated the antiplatelet effect and pharmacokinetic profile of low-dose ticagrelor in healthy Chinese volunteers. Thirty healthy subjects were randomized to receive standard-dose ticagrelor (180-mg loading dose, 90-mg twice daily [bid] [n = 10]), low-dose ticagrelor (90-mg loading dose, 45-mg bid [n = 10]), or clopidogrel (600-mg loading dose, 75-mg once daily [n = 10]). Platelet reactivity was assessed by using the VerifyNow P2Y12 assay at baseline and 0.5, 1, 2, 4, 8, 24, 48, and 72 hours post-dosing. The ticagrelor and AR-C124910XX concentrations were measured for pharmacokinetic analysis. The percentage inhibition of P2Y12 reaction units was higher in the low-dose and standard-dose ticagrelor group than in the clopidogrel group at 0.5, 1, 2, 4, 8, and 48 hours post-dosing (*P* < 0.05 for all), but did not differ significantly between the two ticagrelor doses at any time-point (*P *> 0.05). The plasma ticagrelor and ARC124910XX concentrations were approximately 2-fold higher with standard-dose versus low-dose ticagrelor. No serious adverse events were reported. In conclusion, low-dose ticagrelor achieved faster and higher inhibition of platelet functions in healthy Chinese subjects than did clopidogrel, with an antiplatelet efficacy similar to that of standard-dose ticagrelor.

Despite advances in its diagnosis and management, coronary artery disease is the leading cause of morbidity and mortality worldwide^1^. A combination of aspirin and clopidogrel is the standard therapy for the prevention of recurrent cardiovascular events in patients with acute coronary syndromes (ACS)^2^,^3^,^4^. Nevertheless, there have been several limitations in the uses of clopidogrel^5^, including delayed onset of action^6^, considerable interindividual variability in the platelet response^7^, and poor and irreversible inhibition of platelet aggregation (IPA)^8^.

Ticagrelor is an oral, direct-acting, reversible P2Y12 receptor antagonist that inhibits ADP-induced platelet aggregation^9^,^10^. Unlike the thienopyridines clopidogrel, ticagrelor does not require activation via CYP450 enzymes^11^ and its active metabolite (AR-C124910XX) also has a similar potency in inhibiting the P2Y12 receptor^9^,^12^. In comparative trials, ticagrelor has been reported to exhibit greater and more rapid platelet inhibition than clopidogrel^9^,^10^,^11^,^12^,^13^. Moreover, in the PLATelet inhibition and patient Outcomes (PLATO) trial, ticagrelor (180-mg loading dose, 90-mg twice daily [bid]) has been found to be superior to clopidogrel (300 to 600-mg loading dose, 75-mg once daily [qd]) in reducing cardiac events without causing a significant increase in the incidence of bleeding^14^. However, only 416 Chinese patients were included in this trial, and increasing evidence suggests the existence of different antiplatelet efficacy and safety profiles between Asian and Caucasian subjects^1^,^15^. Notably, our recent study has found that the mean P2Y12 reaction units (PRU) value in Chinese patients after ticagrelor treatment (90-mg bid) is far below the previous thresholds associated with a bleeding risk^16^. Additionally, previous studies have suggested that exposure to ticagrelor and AR-C124910XX is higher in Asian than in Caucasian subjects^15^,^17^.

These findings raise a clinical question as to whether the current recommended dose of ticagrelor is suitable for the Chinese population. Therefore, we conducted this study to assess the antiplatelet effect and pharmacokinetic profile of low-dose ticagrelor (90-mg loading dose followed by a 45-mg bid maintenance dose [MD]) in healthy Chinese volunteers.

## Methods

### Study population

Healthy male and female (non-pregnant, surgically sterile, or post-menopausal) Chinese volunteers between 18 and 45 years of age were enrolled in the study. The subjects were eligible to participate if they had a body mass index (BMI) between 18.5 and 24 kg/m^2^ and a body weight >50 kg, and were in good health according to their medical history, physical examination, vital signs, laboratory tests, and 12-lead electrocardiography (ECG). The exclusion criteria were as follows: (1) haemoglobin <120 g/L for male and <110 g/L for females; (2) platelet count <100 × 10^9^/L; (3) use of anticoagulant or antiplatelet agents or nonsteroidal anti-inflammatory drugs within 2 weeks before enrollment; and (4) a history or presence of conditions known to interfere with the absorption, metabolism, or excretion of drugs. All subjects provided written informed consent, and the study was performed in accordance with the principles established in the Declaration of Helsinki and in compliance with the ICH/Good Clinical Practice. All experimental protocols were approved by the Ethics Committee of Changhai Hospital, Second Military Medical University.

### Study design and treatments

**Figure 1:**
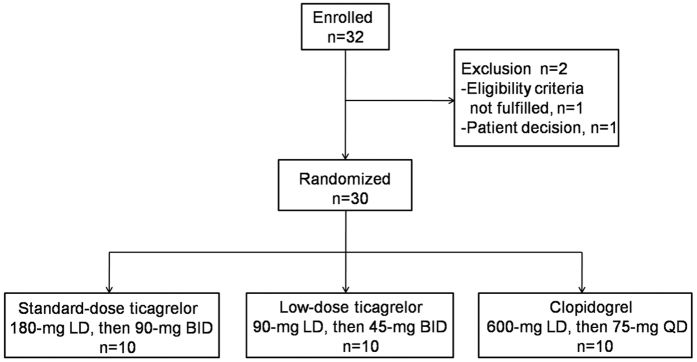
Flow chart. LD, loading dose; BID, twice daily; QD, once daily.

This study was a phase I, single-center, open-label, randomized, controlled study performed to examine the antiplatelet effect and pharmacokinetic profiles of low-dose ticagrelor. The trial was registered at the Chinese Clinical Trial Registry. The trial registration No. was ChiCTR-IPR-15006505 and the date of registration was June 5, 2015. A flow chart diagram of the study is shown in Fig. 1. The randomization was stratified on the basis of sex. The subjects were randomized 1:1:1 to receive standard-dose ticagrelor (180-mg loading dose and then 90-mg bid starting 12 h post-loading), low-dose ticagrelor (90-mg loading dose then 45-mg bid starting 12 h post-loading), or clopidogrel (600-mg loading dose then 75-mg qd). Ticagrelor 90-mg tablets were provided by AstraZeneca and clopidogrel 75-mg tablets were purchased from Sanofi-Aventis. The ticagrelor 90-mg tablet was evenly divided into halves (each containing 45 mg of ticagrelor) by using a pill cutter. The subjects were administered the morning dose after a 10 h overnight fast and the evening dose at least 1 h prior to a meal (with approximately 150 ml of water).The final dose of the study drug was administered on the fourth morning. On day 1, the subjects were admitted into the phase I clinical ward and they stayed in the ward until discharge on day 7.

### Pharmacodynamic analysis

Blood samples for the platelet function analysis were collected at 0 (pre-dose), 0.5, 1, 2, 4, 8, 24, 48, and 72 h after being loaded into 2 vacuum tubes (Becton-Dickinson, Franklin Lakes, NJ, USA) containing 3.2% trisodium citrate. The first tube was discarded to avoid spontaneous platelet activation. The second tube was gently inverted 3 times to ensure complete mixing of the anticoagulant and blood sample and then left to stand for at least 10 minutes at room temperature before analysis. The blood sample was analyzed within 2 h for the rapid platelet-function assay.

Platelet activity was analyzed using VerifyNow P2Y12 (Accumetrics, San Diego, CA, USA). The results are reported in P2Y12 reaction units (PRUs), baseline values (BASEs), and percentage inhibition. The percentage inhibition was calculated as follows: ([BASE-PRU]/BASE) × 100. A PRU > 208 was defined as high on-treatment platelet reactivity (HTPR), which is related to a higher risk of an ischemic event; a PRU < 85 was defined as low on-treatment platelet reactivity, which is related to a higher risk of a bleeding event. The technical details of the platelet function assay have been previously described^17^.

### Pharmacokinetic analysis

The pharmacokinetics of ticagrelor and ARC124910XX were assessed from blood samples taken pre-dose and post-dose at 0.5, (0.75), 1, (1.5), 2, 4, (6), 8, (12), 24, 48, 72, 72.5, 73, 74, 75, 76, 77, 78, 80, 84, 88, 96, 108, 120, and 144 h after loading (time points in brackets used the interval-time sampling method). The platelet function and pharmacokinetic were performed pre-dose and throughout the onset period (0.5 to 72 hours after the first loading dose); after the last dose (at 72 hours), the pharmacokinetic analysis was performed throughout the offset period (72.5 to 144 hours after the first loading dose). Blood samples were collected into vacuum tubes with lithium-heparin and centrifuged at 5000 rpm for 10 min within 60 min of collection. The resultant plasma was transferred to a plain polypropylene tube and stored frozen at −80 °C prior to the analysis.

The ticagrelor and AR-C124910XX plasma concentrations were quantified using liquid chromatography-tandem mass spectrometry (LC-MS/MS) after protein precipitation. For ticagrelor and ARC124910XX, the lower limits of quantification were 40.0 ng/mL (range 40.0–1600.0 ng/mL) and 27.7 ng/mL (range 27.7–1108.0 ng/mL), the intra-batch accuracy was 91.9–109.0% and 86.8–109.2%, and the intra-batch precision was 4.0–8.4% and 5.2–16.9%, respectively.

The pharmacokinetic parameters were calculated by using standard noncompartmental methods with WinNonlin Professional (Version. 4.1; Pharsight Corporation, Palo Alto, CA, USA). The primary pharmacokinetic parameters included the peak plasma concentration (C_max_), minimum plasma concentration (C_min_), time to reach C_max_ (t_max_), terminal-phase half-life (t_½_), area under the plasma concentration-time curve (AUC) from time 0 to infinity (AUC_0-∞_), and the AUC from time zero to the final measurable time-point (AUC_0-t_; calculated by using the linear trapezoidal rule). An inhibitory effect sigmoid maximum observed plateau effect (E_max_) model was used to compare the percentage inhibition of PRU and the pharmacokinetic relationship in healthy Chinese subjects as follows:


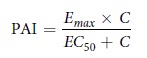


E_max_ = maximum effect, EC_50_ = concentration producing 50% of the maximum effect, and C = plasma concentration.

### Safety and Tolerability

Safety was assessed throughout the study, including the incidence and severity of adverse events (bleeding, arrhythmias, and dyspnea), clinical laboratory evaluations, vital signs, physical examinations and 12-lead ECG. Bleeding events (major, minor, or minimal) were assessed according to the Thrombolysis in Myocardial Infarction [TIMI] criteria.

### Statistical analysis

Continuous variables are expressed as the mean ± standard deviation (SD) or median (interquartile range) as appropriate and categorical variables are presented as percentages. Continuous variables were compared with the analysis of variance (ANOVA) or Kruskal-Wallis H test. Categorical variables were compared with the Chi-square test or Fisher’s exact test. Intragroup differences in the percentage inhibition of PRU were compared using the Wilcoxon signed-rank test. Differences between treatment groups were analyzed using the analysis of covariance model with the baseline PRU as a covariate. The analyses were performed using SPSS version 19.0 software (SPSS Inc., Chicago, IL, USA); a two-sided *P* value < 0.05 was considered to be statistically significant.

## Results

### Baseline characteristics

A total of 30 healthy Chinese subjects were randomized to receive standard-dose ticagrelor (180-mg loading dose, 90-mg bid, n = 10), low-dose ticagrelor (90-mg loading dose, 45-mg bid, n = 10), or clopidogrel (600-mg loading dose, 75-mg qd, n = 10). The median age and mean body mass index (BMI) of the study population were 24 years (range 22–34 years) and 21.44 ± 1.86 kg/m^2^, respectively. The demographic, clinical and laboratory characteristics did not differ significantly among the three treatment groups (Table 1).

### Pharmacodynamics assessment

**Figure 2:**
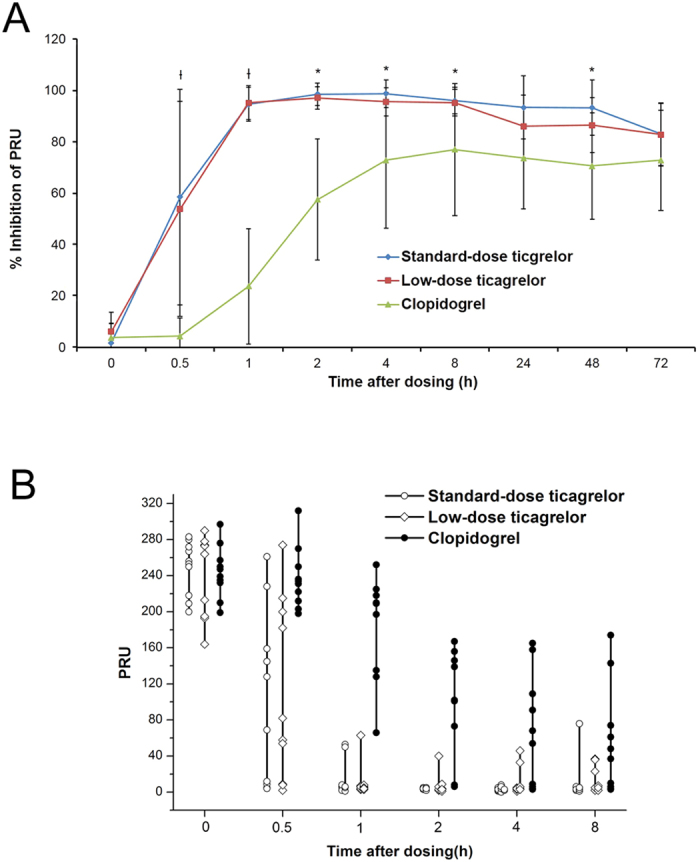
Platelet reactivity as assessed by the VerifyNow P2Y12 assay across time points. (**A**) Mean % inhibition of PRU after LD and MD of standard-dose ticagrelor, low-dose ticagrelor or clopidogrel. ^†^*P* < 0.001, *P < 0.05, low-dose ticagrelor vs clopidogrel. (**B**) The individual PRU values at all time points. LD, loading dose; BID, twice daily; PRU, P2Y12 reaction units.

The mean percentage inhibition of PRU measured by the VerifyNow assay was significantly higher in the low-dose and standard-dose ticagrelor groups than in the clopidogrel group at 0.5, 1, 2, 4, 8, and 48 h after loading (all *P* < 0.05, except 24 and 72 h, *P *> 0.05; Fig. 2A), whereas there was no significant difference in the mean percentage inhibition between the two ticagrelor doses at any time point (*P *> 0.05). At 0.5 h after loading, low-dose and standard-dose ticagrelor (53.9% and 58.5%) achieved a higher mean percentage inhibition than clopidogrel (4.4%, *P* < 0.001). The mean percentage inhibition for both ticagrelor doses peaked 2–4 h after loading compared with the 8 h peak observed for clopidogrel (97.1%, 98.7% vs. 77%, Fig. 2A). To compare inter-individual variability in the response between each group, all individual PRU values are presented in Fig. 2B. At 1 h after loading, a lower variability in the percentage inhibition was observed in both ticagrelor groups, compared with the clopidogrel group.

### First-dosing pharmacokinetics of ticagrelor and AR-C124910XX

**Figure 3:**
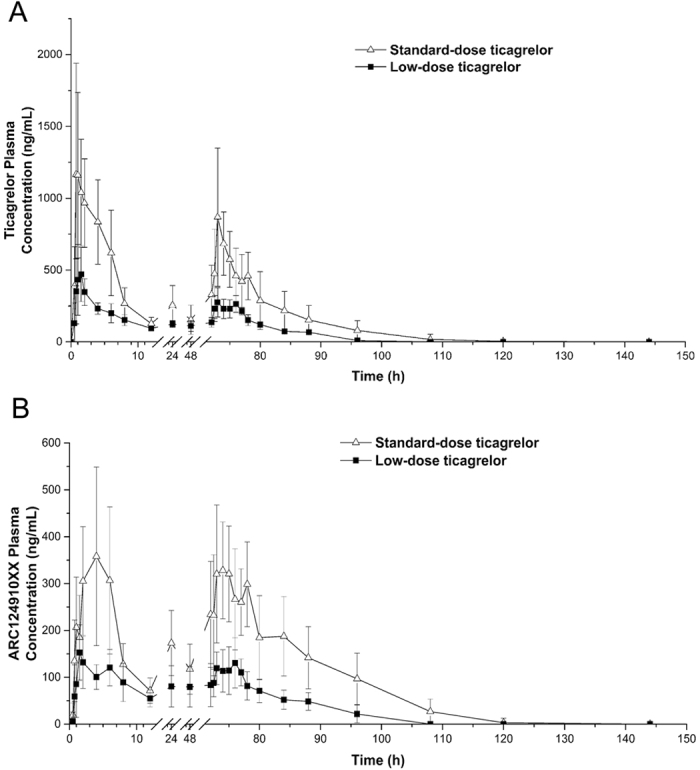
Mean plasma concentrations of ticagrelor (**A**) and AR-C124910XX (**B**) across time points.

Ticagrelor was rapidly absorbed after a single loading dose of 90 mg or 180 mg (Fig. 3A), with a median t_max_ of 1.25 and 1.75 h (Table 2), respectively. The major metabolite ARC124910XX (Fig. 3B) was also rapidly formed after ticagrelor loading, with a median t_max_ of 2 h (Table 2). The T_max_ values of ticagrelor and AR-C124910XX were independent of the ticagrelor dose. The C_max_ and AUC_0-t_ values of ticagrelor and ARC124910XX after loading with 180-mg of ticagrelor were approximately 2–3-fold higher than the values obtained with 90 mg of ticagrelor. Exposure to AR-C124910XX was approximately 30–40% of the exposure to ticagrelor.

### Last-dosing pharmacokinetics of ticagrelor and AR-C124910XX

The absorption of ticagrelor and the conversion to AR-C124910XX was rapid after administration of ticagrelor 90-mg bid or 45-mg bid (Fig. 3A). At steady state (day 4), the median t_max_ was 1 h for ticagrelor 90-mg bid versus 2 h for 45-mg bid, and the mean t_1/2_ was 8.9 h versus 8.4 h, respectively (Table 2). For AR-C124910XX (Fig. 3B), the median t_max_ was 1.5 h for ticagrelor 90-mg bid versus 4 h for 45-mg bid and the mean t_1/2 _was 11.7 h versus 16.6 h, respectively (Table 2). The exposure to ticagrelor and AR-C124910XX was proportional to the ticagrelor dose, because the mean C_max_ and AUC_0–∞_ values for both analytes were approximately 2–3-fold higher for ticagrelor 90-mg bid than for ticagrelor 45-mg bid.

### Pharmacokinetic/antiplatelet effect relationship

**Figure 4:**
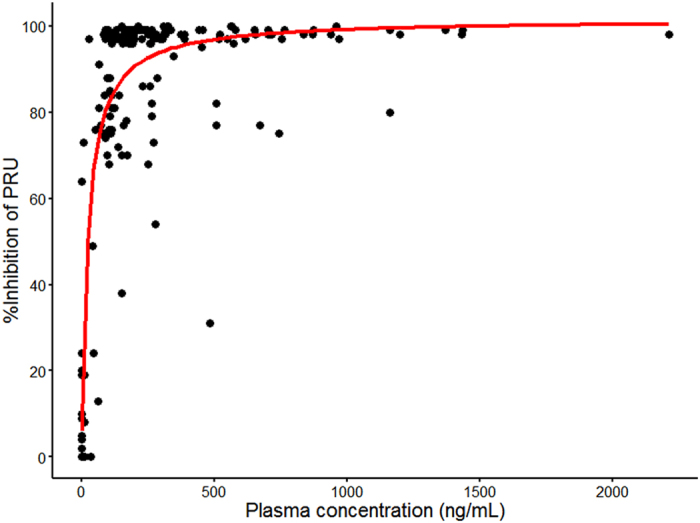
Ticagrelor plasma concentration versus % inhibition of PRU. PRU, P2Y12 reaction units.

As shown in Fig. 4, the percentage inhibition of PRU increased with increasing ticagrelor plasma concentrations. Using pooled data, the E_max_ estimate was 100% and the estimate for the 50% E_max_ was 24.5 ng/mL, which indicated that, as the doses increased, the ticagrelor plasma concentrations were sufficiently high to achieve and sustain a high IPA in Chinese healthy subjects.

### Safety and Tolerability

Both ticagrelor doses were generally well tolerated, and no severe adverse events occurred during the study. Three subjects (2 under standard-dose ticagrelor treatment and 1 under low-dose ticagrelor treatment) reported minimal bleeding, and 1 subject (under clopidogrel treatment) exhibited sinu-bradycardia. No subjects discontinued the study treatment due to adverse events.

## Discussion

This study is the first to assess the antiplatelet effect and pharmacokinetic profile of low-dose ticagrelor (90-mg loading dose, 45-mg bid) in healthy Chinese subjects. The major findings of the present study are as follows: (1) both standard-dose and low-dose ticagrelor exhibited faster and greater inhibitory effects and less variability in the IPA than clopidogrel in this population; (2) the mean percentage inhibition of PRU measured by VerifyNow P2Y12 was numerically but not significantly lower with standard-dose ticagrelor than with low-dose ticagrelor; and (3) the ticagrelor and ARC124910XX plasma concentrations were approximately 2-fold higher for standard-dose than low-dose ticagrelor.

There is a higher prevalence of CYP2C19 polymorphisms and HTPR in the Chinese population compared with the white population despite clopidogrel use^16^,^18^. This higher prevalence may be associated with an increased risk of recurrent cardiovascular events. Ticagrelor has a more rapid and greater antiplatelet efficacy with an improved prevention of clinical thrombotic events in patients with ACS, as compared with clopidogrel^6^,^14^. However, the benefit of ticagrelor relative to clopidogrel is accompanied by a higher bleeding risk^14^,^19^. In particular, in the PLATO trial, ticagrelor was found to be associated with a higher rate of non-coronary artery bypass graft-related bleeding, although no significant difference in the rate of overall major bleeding were found between the ticagrelor and clopidogrel groups^14^. Additionally, in a Chinese subgroup analysis of PLATO^14^ the rate of major bleeding in the ticagrelor group was numerically higher than the rate in the clopidogrel group (6.8% vs. 3.9%), although the difference is not significant, possibly because of the limited sample size. Our recent study has demonstrated that ticagrelor (180-mg loading dose, 90-mg bid) is significantly more effective than high-dose clopidogrel in overcoming HTPR; however, ticagrelor results in a very low platelet reactivity level (44.38 ± 40.26 PRU), thus indicating a potentially heightened risk for bleeding^16^. Therefore, it is important to increase the knowledge base for determining the optimal ticagrelor dosing strategy, particularly in Chinese people who may be more vulnerable to bleeding complications^20^,^21^,^22^.

### Pharmacokinetics

In studies with healthy volunteers, ticagrelor absorption has been found to occur rapidly after oral administration and exposure to ticagrelor and AR-C124910XX has been found to increase in a dose-dependent manner, indicating linear pharmacokinetics^23^. Consistently with previous results, our pharmacokinetic data showed that the absorption of ticagrelor and conversion to AR-C124910XX were rapid and at steady state with ticagrelor 90-mg bid, the Cmax and systemic exposure (AUC_0–∞_) of ticagrelor and AR-C124910XX were approximately 2-fold higher than those obtained with ticagrelor 45-mg bid.

According to previous study results, Asian subjects have higher exposure to ticagrelor and ARC124910XX than Caucasians^15^,^17^. Li *et al*.^17^ have reported that exposure to ticagrelor and the AR-C124910XX is approximately 40% higher in Chinese volunteers than in volunteers of Caucasian ethnicity. Consistently with these findings, in the current study the AUC_0–∞_ and C_max_ of ticagrelor and AR-C124910XX at steady state were approximately 30–50% higher than the previously reported observations in healthy Caucasian subjects and patients with atherosclerosis and ACS.

### Pharmacodynamics

Unlike the thienopyridines, ticagrelor is an active agent that does not require hepatic biotransformation; this characteristic may account for the rapid onset of its antiplatelet effect^12^,^23^. In the DISPERSE and DISPERSE-2 studies, a maximal antiplatelet effect occurred 2 h after loading with ticagrelor^24^,^25^. In the ONSET/OFFSET study^6^, the onset of the antiplatelet effect of ticagrelor occurred within approximately 30 min and to be markedly greater for ticagrelor than clopidogrel 2 h after loading (88% vs. 38%, *P* < 0.0001). In the present study, a significant antiplatelet effect was observed at 0.5 h after administration of a 90-mg or 180-mg loading dose of ticagrelor and the peak IPA occurred 2–4 h post-dose compared with 8 h in the clopidogrel group. Furthermore, the magnitude of the percentage inhibition of PRU in the current study (standard-dose: 98.7% and low-dose 97.1%) was higher than in the ONSET/OFFSET study (approximately 83%)^6^, thus indicating that the response to ticagrelor is variable in different populations.

The antiplatelet effect of ticagrelor was dose-related, with an increasing dose enhancing the response^23^. The DISPERSE study^24^ has demonstrated that ticagrelor (100–200-mg bid and 400-mg qd) achieves a more rapid and greater steady-state IPA than ticagrelor 50-mg bid or clopidogrel 75-mg qd. However, in our study, the magnitude of platelet inhibition and variability did not differ significantly between the low-dose and standard-dose ticagrelor groups, despite the half-dose administration. One explanation for this phenomenon may be the high exposure to ticagrelor and AR-C124910XX in Asian subjects. Importantly, the results from the present study are in line with the observations in the PEGASUS-TIMI 54^19^ and a substudy^26^ in which the low-dose ticagrelor had a level of antiplatelet efficacy similar to the 90-mg bid does and to significantly reduce the risk of cardiovascular events in patients with prior myocardial infarction in spite of ticagrelor plasma concentration being approximately 1/3 lower with low-dose ticagrelor (60 mg vs. 90 mg). Moreover, similar results have been observed in two recent studies on low-dose ticagrelor in Asian populations^27^,^28^. In a randomized trial of healthy Korean subjects^27^, a 90-mg loading dose and 90-mg qd of ticagrelor was found to induce more rapid and potent inhibition of platelet function than clopidogrel (600-mg loading dose and 75-mg MD). Moreover, Japanese and Asian patients with stable coronary artery disease (CAD), ticagrelor 45-mg bid and 90-mg bid were found to be associated with a greater final-extent IPA than clopidogrel 75-mg^28^. Our results confirmed these results and demonstrated that low-dose ticagrelor achieves more potent antiplatelet efficacy than clopidogrel.

### Safety

During ticagrelor therapy, adverse events such as dyspnea, bleeding, and ventricular pauses occur frequently and lead to a high rate of drug discontinuation^14^,^29^. An ideal dosing regimen would be adequate to produce the clinical benefits of increased platelet inhibition with minimal side effects^30^. Previous studies have reported that the frequency of adverse events related to ticagrelor is associated with a dose-dependent increase^9^,^31^,^32^. Remarkably, in PEGASUS-TIMI 54^19^ and a substudy^26^, ticagrelor 60-mg had a similar level of antiplatelet efficacy as 90-mg bid. However, the rates of bleeding and dyspnea leading to discontinuation of the study drug were numerically lower with the 60-mg dose of ticagrelor than with the 90-mg dose, thus suggesting a better safety profile with the 60-mg dose. In this study, no major bleeding and dyspnea events were observed for the two ticagrelor doses, whereas the rate of minimal bleeding was numerically higher with the standard-dose than with the low-dose ticagrelor. These findings show that low-dose ticagrelor may offer efficacy and safety similar to standard-dose ticagrelor.

### Limitations

The present study has some limitations. Lower doses of ticagrelor (<45 mg) were not assessed in the current study, but no marked IPA was observed at ticagrelor doses <30 mg^33^. The platelet reactivity in the present study was evaluated only with the VerifyNow P2Y12 assay. However, there is a poor correlation among currently available point-of care assays^34^, and the VerifyNow P2Y12 assay is the most widely accepted test for assessing the antiplatelet effects of P2Y12 receptor inhibitors^35^,^36^. Additionally, our study was conducted in a small number of young healthy subjects with a short duration of treatment and not in patients with CAD, who may not have yielded similar findings.

## Conclusion

In healthy Chinese subjects, low-dose ticagrelor produced an antiplatelet efficacy similar to that of standard-dose ticagrelor, which was faster and more potent than the effect of clopidogrel. These results have important clinical implications for the selection of a suitable antiplatelet dosing strategy for Chinese subjects and will be explored in a large-scale randomized clinical trial in CAD patients.

## Additional Information

**How to cite this article**: Li, P. *et al*. Low-dose ticagrelor yields an antiplatelet efficacy similar to that of standard-dose ticagrelor in healthy subjects: an open-label randomized controlled trial. *Sci. Rep.* **6**, 31838; doi: 10.1038/srep31838 (2016).

## Figures and Tables

**Table 1:** Baseline characteristics.

	Standard-dose ticagrelor (n = 10)	Low-dose ticagrelor (n = 10)	Clopidogrel (n = 10)	*P*-Value
Age, years	24.0 (24.0–25.3)	24.5 (22.8–26.0)	24.5 (23.8–25.50)	0.969
Male gender, n (%)	5 (50)	5 (50)	5 (50)	1.000
BMI, kg/m^2^	21.7 ± 1.7	21.2 ± 2.2	21.4 ± 1.8	0.815
RBC, ×10^12^/L	4.7 ± 0.47	4.8 ± 0.5	5.0 ± 0.7	0.341
WBC, ×10^9^/L	6.3 (5.1–7.6)	7.2 (5.3–8.8)	5.9 (5.7–7.3)	0.552
Hemoglobin, g/L	133.5 (128.0–147.5)	135.0 (120.0–160.5)	143.50 (128.0–153.3)	0.729
Mean platelet volume, fl	9.4 (8.8–10.5)	9.5 (9.1–9.8)	9.2 (8.4–9.5)	0.347
Platelet count, ×10^9^/L	237.8 ± 56.5	210.9 ± 41.1	230.6 ± 52.0	0.474
Uric acid, umol/L	0.31 ± 0.08	0.33 ± 0.07	0.33 ± 0.07	0.733
Creatinine, umol/L	75.1 ± 13.5	67.0 ± 11.3	73.3 ± 16.5	0.407
Fasting serum glucose, mmol/L	4.84 ± 0.39	5.01 ± 0.30	5.00 ± 0.53	0.598
ALT, U/L	11.5 (8.8–14.3)	12.5 (10.8–18.3)	14.0 (10.8–16.3)	0.413
AST, U/L	17.5 (16.8–19.0)	17.0 (15.0–20.3)	18.5 (16.8–20.5)	0.488
VERIFYNOW-P2Y12 PRU	254.5 (215.8–274.0)	238.5 (193.0–275.0)	243.0 (226.5–261.8)	0.736
VerifyNow BASE	233.6 ± 17.1	234.1 ± 28.8	247.6 ± 26.6	0.369
VerifyNow% inhibition	0 (0–2.0)	3.0 (0–11.5)	0 (0–10.5)	0.153

Values are mean  ±  SD, n (%), or median (interquartile range); BMI = body Mass Index, PRU = P2Y12 reaction units; WBC = white blood cell; RBC = red blood cell; ALT = Alanine aminotransferase; AST = aspartate aminotransferase.

**Table 2:** Pharmacokinetic parameters for ticagrelor and AR-C124910XX following the first-dosing and last-dosing.

Parameter	First-dosing		Last-dosing	
	ticagrelor 180-mg (n = 10)	ticagrelor 90-mg (n = 10)	ticagrelor 90-mg bid (n = 10)	ticagrelor 45-mg bid (n = 10)
**Ticagrelor**				
C_max_, ng/mL^a^	1447 (27)	470 (48)	921 (46)	322 (18)
AUC_0-t_, ng·h/mL^a^	5923 (25)	2158 (22)	7110 (60)	2420 (19)
AUC_0-∞_, ng·h/mL^a^	6905 (30)	3330 (22)	8023 (55)	3128 (18)
t_max_, h^b^	1.75 (0.75–4.0)	1.25 (0.75–2.0)	1.00 (1.0–2.0)	2.0 (1.0–5.0)
t_1/2_, h^a^	NC	NC	8.9 (31)	8.4 (37)
Accumulation ratio^c^			1.7 (18)	1.6 (25)
**AR-C124910XX**				
C_max_, ng/mL^a^	445 (35)	154 (39)	361 (35)	142 (35)
AUC_0-t_, ng·h/mL^a^	2120 (34)	887 (25)	4778 (45)	1337 (36)
AUC_0-∞_, ng·h/mL^a^	2836 (31)	2782 (62)	6407 (46)	2467 (33)
t_max_, h^b^	2.0 (1.5–6.0)	2.0 (1.0–4.0)	1.5 (1.0–6.0)	4.0 (1.0–5.0)
t_1/2_, h^a^	NC	NC	11.7 (32)	16.6 (54)
Accumulation ratio^c^			2.0 (20)	2.6 (42)

^a^Values are geometric mean (% coefficient of variation).

^b^Values are median (range).

^c^Accumulation ratio = 1/(1−exp(−elimination phase slope *12)).

NC: Due to the impact of the maintenance dose starting 12 h post-dosing, the pharmacokinetic parameters on the first day did not include all the clearance rate information and thus the t_1/2_ value of ticagrelor and AR-C124910XX on the first day was not given. Cmax = maximum plasma concentration; AUC_0-t_ = area under the plasma concentration-time curve from time zero to the final measurable time-point; AUC_0-∞_ = area under the plasma concentration-time curve from time zero to infinity; t_max_ = time to reach C_max_; t_1/2_ = elimination half-life; bid = twice daily.
